# Skin Color and Severe Maternal Outcomes: Evidence from the Brazilian Network for Surveillance of Severe Maternal Morbidity

**DOI:** 10.1155/2019/2594343

**Published:** 2019-07-30

**Authors:** K. G. Fernandes, M. L. Costa, S. M. Haddad, M. A. Parpinelli, M. H. Sousa, J. G. Cecatti, the Brazilian Network for Surveillance of Severe Maternal Morbidity Study Group

**Affiliations:** ^1^Department of Obstetrics and Gynaecology, University of de Campinas (UNICAMP), School of Medicine, Campinas, São Paulo, Brazil; ^2^Jundiai School of Medicine, Jundiaí, São Paulo, Brazil; ^3^The Coordinating Institution, The Obstetric Unit from the School of Medical Sciences, University of Campinas, Brazil

## Abstract

**Background:**

Taking into account the probable role that race/skin color may have for determining outcomes in maternal health, the objective of this study was to assess whether maternal race/skin color is a predictor of severe maternal morbidity.

**Methods:**

This is a secondary analysis of the Brazilian Network for Surveillance of Severe Maternal Morbidity, a national multicenter cross-sectional study of 27 Brazilian referral maternity hospitals. A prospective surveillance was performed to identify cases of maternal death (MD), maternal near miss (MNM) events, and potentially life-threatening conditions (PLTC), according to standard WHO definition and criteria. Among 9,555 women with severe maternal morbidity, data on race/skin color was available for 7,139 women, who were further divided into two groups: 4,108 nonwhite women (2,253 black and 1,855 from other races/skin color) and 3,031 white women. Indicators of severe maternal morbidity according to WHO definition are shown by skin color group. Adjusted Prevalence Ratios (PR_adj_ - 95%CI) for Severe Maternal Outcome (SMO=MNM+MD) were estimated according to sociodemographic/obstetric characteristics, pregnancy outcomes, and perinatal results considering race.

**Results:**

Among 7,139 women with severe maternal morbidity evaluated, 90.5% were classified as PLTC, 8.5% as MNM, and 1.6% as MD. There was a significantly higher prevalence of MNM and MD among white women. MNMR (maternal near miss ratio) was 9.37 per thousand live births (LB). SMOR (severe maternal outcome ratio) was 11.08 per 1000 LB, and MMR (maternal mortality ratio) was 170.4 per 100,000 LB. Maternal mortality to maternal near miss ratio was 1 to 5.2, irrespective of maternal skin color. Hypertension, the main cause of maternal complications, affected mostly nonwhite women. Hemorrhage, the second more common cause of maternal complication, predominated among white women. Nonwhite skin color was associated with a reduced risk of SMO in multivariate analysis.

**Conclusion:**

Nonwhite skin color was associated with a lower risk for severe maternal outcomes. This result could be due to confounding factors linked to a high rate of Brazilian miscegenation.

## 1. Background

Significant progress in reducing maternal mortality has been achieved in the past 15 years, in addition to a growing awareness about the burden of severe maternal morbidity. Standard definitions of potentially life-threatening conditions (PLTC) and maternal near miss (MNM) events have enabled our understanding of different conditions and delays related to the quality of maternal health (Box 1 provides the full list of WHO criteria for both conditions) [1–5].

The identification of risk factors can be effective in providing adequate prevention and surveillance of women with severe maternal morbidity, allowing early diagnosis and treatment of complications [1]. It is essential that global disparities are acknowledged among high-income and low-income settings to improve health care. Ethnic or racial inequalities also require investigation within nations [6, 7].

In general, white women have better maternal health outcomes [6] while black women usually have higher maternal mortality ratios [8]. It is more likely that black women are younger and lack a partner [6]. Furthermore, these women usually have a lower level of school education and less adequate prenatal care assistance [5, 8]. Recent data on severe maternal morbidity and different races also confirmed that black women have higher chances of developing MNM events [9]. Other minority ethnic groups have been investigated and associated with worse maternal outcomes within nations, such as White-Hispanics in New York, USA [10], and indigenous populations worldwide [11]. Not only race/skin color, but also other socioeconomic and demographic factors, preexisting conditions, and pregnancy complications are also associated with poor maternal and perinatal outcomes [12, 13].

Another relevant challenge in studying race/ethnicity lies in the extensive admixture of a population, for example, in a country like Brazil, where this information is clearly difficult to collect and interpret [14]. Therefore, our aim was to explore data on skin color variations and maternal morbidity from the Brazilian Network for Surveillance of Severe Maternal Morbidity study.

## 2. Methods

This study is a secondary data analysis from the Brazilian Network for Surveillance of Severe Maternal Morbidity, a multicenter cross-sectional study including 27 referral maternity hospitals (private, public, university, nonuniversity) in Brazil that had at least 1000 deliveries annually [15]. Briefly, the study was conducted from July 2009 to June 2010. A prospective surveillance was carried out to identify cases of maternal death (MD), maternal near miss (MNM), and potentially life-threatening conditions (PLTC), according to standard World Health Organization (WHO) definition and criteria (Box 1) [1]. Other particularities of the study methodology and main results have been previously published elsewhere [2, 16].

Sample size was calculated based on a previous pilot study that obtained a MD ratio of 70/100,000 live births and a MNM ratio estimated as maximum 20% of the severe maternal morbidity rate of 42/1000 live births [17]. Thus, the expected prevalence of 8 MNM/1000 births with a 95% confidence interval indicated that a surveillance of 75 thousand deliveries was required to achieve at least 100 MD and 600 MNM.

Data collection was performed by investigators and research assistants allocated to each hospital. Medical charts of all women who fulfilled inclusion criteria were reviewed after hospital discharge or death. If there was any doubt about a diagnosis or procedure, the physician responsible for the case was sought for elucidation. For each case identified, a pretested and coded form was used. The form included 80 items, e.g., data on sociodemographic and economic characteristics; obstetric history; prenatal, childbirth, and postpartum periods; neonatal outcome, complications, and delays in medical care.

Data was entered in the OpenClinica® - version 2.5.5 - (Akaza Research, Waltham, MA, USA) electronic platform. The current article is an analysis of the occurrence of severe maternal morbidity among different ethnic/skin color categories. The study was approved by the National Research Ethics Council (CONEP) and Institutional Review Board of the participating locations. No contact was maintained with participants, only chart review; therefore a waiver of informed consent term was granted. The National Scientific Technological Development Council (CNPq) and the Department of Science and Technology of the Ministry of Health (DECIT) funded the study.

For the current analysis, data on ethnic/race/skin color variations were further explored. In the form, according to information retrieved from the medical chart and self-reported, the skin color was categorized as white, black, indigenous, yellow, and others. In the study, there were 31.7% of white women, 23.6% of black women, 0.2% of indigenous women, 0.3% of yellow women, 19.0 % of others (mainly those from a mixed group of black and white, known as* pardo*), and 25.3% of missing data. Although the rate of missing data appears to be relatively high, it was distributed equally between the groups and was due to lack of information in the clinical records. Due to the small number of indigenous, yellow, and other ethnicities, we chose to group these women along with the black ethnic group, representing the nonwhite group. Therefore, analysis was further conducted in two groups: whites and nonwhites (black, indigenous, yellow, and others).

First, we demonstrated the indicators of severe maternal morbidity defined by the WHO (Maternal Near Miss Ratio, Severe Maternal Outcomes Ratio, Maternal Near Miss to Maternal Mortality ratio, and Mortality Index) according to ethnic group. Next, we evaluated the distribution of pregnancy outcomes (degrees of maternal morbidity) according to the main causes of obstetric complications and skin color, using *χ*^2^ and Fisher's exact test to evaluate any differences between groups. Furthermore, the risk of severe maternal outcome (SMO = MNM + MD) was estimated by skin color group, according to sociodemographic characteristics, obstetric characteristics, pregnancy outcome, and perinatal results (PR adj - 95%CI). Multiple analysis (Poisson regression) was used to assess conditions independently associated with SMO (MNM or MD) resulting from pregnancies with severe maternal morbidity, including also skin color as a predictor. Data analysis was conducted by using SPSS software version 23 (IBM, Armonk, NY, EUA) and Stata software version 11.2 (StataCorp, College Station, TX, EUA).

## 3. Results

**Figure 1:**
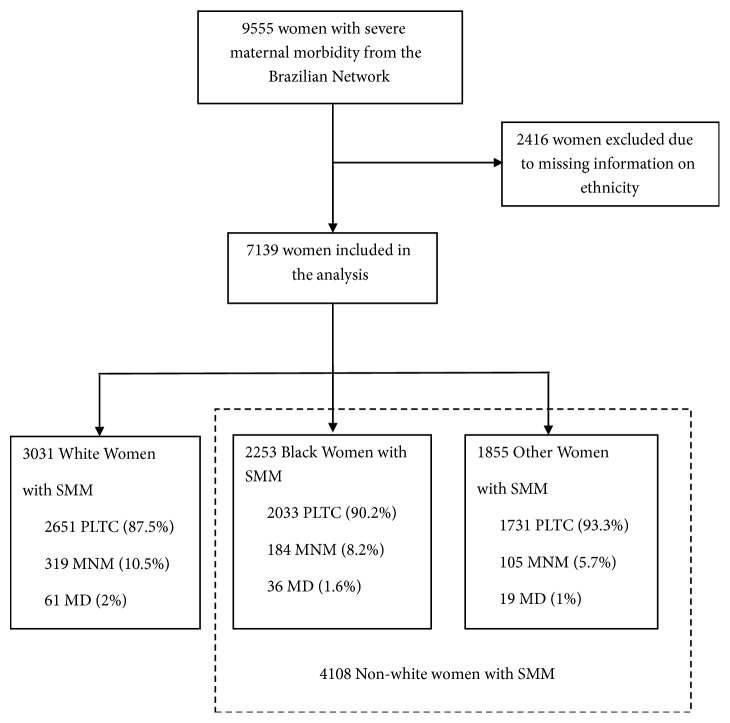
Flow chart of women included in the study.

During the study period, 82,388 women underwent surveillance in 27 participating hospitals, resulting in 82,144 live births. Severe pregnancy-related complications occurred in 9,555 women. Data on skin color was available for 7,139 women (missing data: 25%). All 7,139 women were further divided into major skin color groups: white, black, and others, according to the severity of morbidity (Figure 1). The majority of cases were described as white (42.5%), followed by black (31.6%) and others (25.9%). For further analysis of skin color variations, two groups were considered: (1) a group of white women and (2) a group of nonwhite women (black + others). Considering the continuum of morbidity, there were significant differences among the two groups, with a significantly higher proportion of MNM and MD among white women (Table 1).

Allowing for additional recommended health indicators for maternal morbidity and mortality, there were no statistical differences in the maternal near miss to mortality ratio and mortality index between groups (Table 1).

The distribution of pregnancy outcomes according to major causes of obstetric complications was also evaluated by skin color. It revealed that the nonwhite group had a significantly higher proportion of MD due to hypertension, infection, and combined complications than the white group, despite the lower number of PLTC and MNM cases. For hypertension and clinical/surgical complications (which included the burden of the H1N1 influenza pandemic that occurred during study period), in the continuum of severity total numbers were higher among black women and other minority ethnic groups, with a similar or increased proportion of MD among white women (Table 2).

In order to deeply understand factors associated with a worse maternal outcome within the continuum of severity, we further compared cases of SMO (MNM+MD) to cases of PLTC (less severity) among the skin color groups previously defined. This comparison was performed for previous clinical conditions, sociodemographic characteristics, and maternal and perinatal results. Both groups showed significant increases in the prevalence of SMO cases among previous cardiac and thyroid diseases. Women from the nonwhite group had less previous chronic hypertension and obesity, while there was a higher incidence of renal disease, sickle cell disease, and HIV/AIDS among SMO cases of nonwhite group. White women with SMO had a higher prevalence of other relevant conditions: low weight, neurologic disease, cancer, and drug addiction (Table 3).

The risks of SMO were estimated, according to sociodemographic characteristics of women for each skin color group, showing that increased maternal age was the most significant factor associated with severity in both groups. Among the nonwhite group, the lack of a partner was protective. In white women, a higher education was a protection from severity of morbidity. Overweight and obesity reduced the risk of SMO, irrespective of skin color (Table 4). Furthermore, the risks of SMO were estimated according to obstetric characteristics of women for each group, revealing that increased risk occurred among multiparous women (especially parity ≥ 3 in the nonwhite group), inadequate antenatal care, preterm hospital admission, or presence of delays (Table 5) for both groups.

For characteristics of delivery and perinatal outcomes, results were consistently worse (increased risk of severity) among the nonwhite group. There was an overall increased higher risk of preterm deliveries, 5-minute Apgar scores below 7, low birthweight, stillbirth, and neonates with intensive care unit admission or transfer among women with SMO among white compared to nonwhite women. The nonwhite group also had a higher risk of neonatal death among SMO cases (Table 6).

Multiple regression analysis identified conditions independently associated with SMO. The nonwhite group significantly reduced the risk of severity in these women (Table 7).

## 4. Discussion

The current analysis highlights race/skin color variations among cases of maternal morbidity, associated with severity of morbidity. Overall, skin color was not associated with an increased maternal morbidity. The most significant finding was the higher proportion of maternal deaths among the white group, along with increased overall cases of hemorrhage and infection among white women. This group also had worse results in cases of previous chronic hypertension, renal disease, sickle cell disease, and HIV/AIDS and worse perinatal outcomes, when compared to nonwhite women. Nonwhite skin color reduced the risk of SMO in the multivariate analysis of factors independently associated with increased severity.

Before going ahead with these results and their potential implications for the knowledge on this topic, it is necessary to remember the limitations and difficulties we have in obtaining information on ethnicity/skin color in Brazil, first, because according to national rules, the skin color should be autoreported and it is not necessarily recorded in clinical records from all hospitals. This could be an explanation why we had relatively high missing rates for skin color in this study, although similarly distributed according to the severity of the morbid condition. Second, there was a huge miscegenation in Brazil, which made difficult the classification in a specific category.

According to data from the Center for Disease Control and Prevention (CDC), the nonwhite group had more PLTC, in comparison to the white group. However, white women had more severe complications (MNM and MD), and black women had a 4-fold increased chance of dying from pregnancy-related causes compared to white women [18]. In the current study, the maternal mortality ratio was 170.4/100,000 LB, while according to national vital statistics from the same period, the maternal mortality ratio in Brazil was 72/100,000 LB in 2009 and 68.2/100,000 LB in 2010 for the entire country [19]. The 2009 H1N1 influenza A pandemic occurred at the same time period as our study. Maternity hospitals participating in the study are also referral centers; therefore our study had a higher mortality ratio [20].

Other examples from low-income settings present even higher rates of maternal mortality [3, 4, 7]. A study conducted in a hospital in Nigeria showed a maternal mortality ratio of 1908/100 000 LB, which was 11-fold higher than results found in the current study, and a severe maternal outcome ratio of 218/1000 LB, a value that was 20-fold higher than our findings [21].

Underlying social-demographic conditions and previous clinical comorbidities are likely to influence maternal and perinatal outcomes [5, 8]. Low level of school education is considered a risk factor for SMO [5]. In the current study, white women with complete secondary school education had a lower risk of developing SMO. Multiparous nonwhite women had a higher risk of SMO, in accordance with the literature [2].

Regardless of skin color, women of advanced maternal age had a higher risk of developing severe complications (SMO). Similar reports in the literature have described that maternal age over 40 years increases the odds of complications [22–27]. CDC data indicated that black women at advanced maternal age had a higher mortality rate [18]. On the other hand, obesity and overweight were protective factors for SMO.

A previous report on data from Brazil showed that women with underlying disorders had a higher risk of near miss events and nonwhites had more chronic diseases than whites [5]. In our study, the nonwhite group who developed SMO had a lower prevalence of HIV/AIDS and chronic hypertension. In this group, the complication that caused a higher rate of PLTC was mostly hypertension. Nonwhite women have a greater trend towards hypertension diagnosed at the beginning of pregnancy and also of developing preeclampsia [22, 23]. Although white women had more PLTC due to infection, nonwhite women had more severe complications and deaths related to infection, in our sample.

A main concern when considering risk factors for worse outcomes is to identify such conditions and provide adequate care, awareness, and prompt diagnosis of complications, in order to avoid delays. These delays can be organized as related to the delay in deciding to seek care by the individual and/or family (called phase I delay); delay in reaching an adequate health care facility (phase II); and delay in receiving adequate care at the facility (phase III) [15, 28]. The present study shows once more that severe maternal outcome is associated with the occurrence of delays, among nonwhite color and even more among white women.

A study in the United Kingdom showed that starting prenatal care at a later gestational age or no prenatal care may be associated with maternal death in ethnic minority groups [29]. In contrast, a study in Holland did not show any association between late prenatal care or no prenatal care and maternal death [30]. Another study from the United Kingdom showed that pregnancy in black women is diagnosed later, delaying initiation of prenatal care and consequently delaying access to prenatal care [31]. Nevertheless, in our study the lack of prenatal care was a risk factor for SMO among whites. Regardless of ethnicity, the quality of prenatal care is fundamental. On the other hand, women who have many medical prenatal visits usually have some pathological condition or risk justifying this excessive number [5]. Quality assessment of prenatal care is not easy, although there is a suspicion that it may be directly influenced by the social class and ethnic/skin color group of the health service user.

Considering perinatal outcomes, nonwhite group who developed SMO had a higher risk of delivering preterm infants. Furthermore, SMO was associated with a higher occurrence of fetal death, 5-minute Apgar scores lower than 7, low birthweight (<2.500g), need for hospital admission, or transfer of the newborn infant or neonatal death, and these risk estimates were slightly higher for nonwhite than for white women. Two previous Brazilian studies have identified that black women have a higher incidence of low birthweight infants [5, 19].

This network study may have some limitations in determining skin color, since data was captured from medical charts. Information may have been extracted by woman self-report or defined by the medical provider of obstetric care or even the hospital personnel who registered obstetric care management. Furthermore, due to miscegenation in the country, some characteristics that are specific to the black people may be missing, as we observed in some studies conducted in countries with a lower proportion of miscegenation.

In our study, women from the white group were the most affected by SMO, a result that is surprising taking into account all the previously available evidence. We tried to explore the possible interrelationships with other variables that could explain these results, including the difficulty of classifying race/skin color by the women themselves, what could also be seen as a limitation of the study. However, another important point must be also considered: the data came from a prospective surveillance performed during a one-year period in 27 referral maternity hospitals in the country, mainly university tertiary hospitals. We cannot exclude the possibility that a differential access to these facilities was experienced according to race/ethnicity, with higher system and personal difficulties for nonwhite women having access to these more resourceful facilities in dealing with their pregnancy complications. This reflects the importance of studies addressing different risk factors for worse outcomes in low and middle-income settings. Local characteristics, such as significant miscegenation, need to be considered when studying skin color.

## Figures and Tables

**Box 1:**
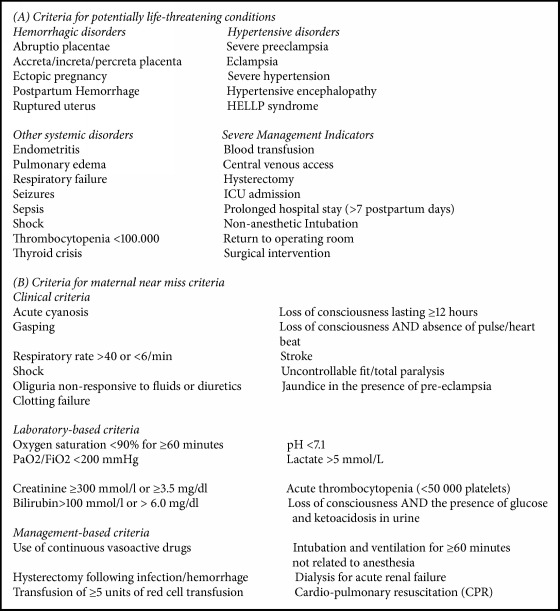
The WHO criteria for potentially life-threatening conditions and maternal near miss (6).

**Table 1:** Indicators of severe maternal morbidity according to the WHO definition by ethnic/skin color groups.

Sample	PLTC	MNM	MD	Total	MNMR^a^	SMOR^a^
Total	8,645 90.4%	770 8.1%	140 1.5%	9,555	9.37/1000 LB	11.08 /1000 LB

With information on ethnicity/skin color^b^	6,415 89.9%	608 8.5%	116 1.6%	7,139	-	-
Non-white	3,764 91.6%	289 7.0%	55 1.3%	4,108	-	-
White	2,651 87.5%	319 10.5%	61 2.0%	3,031	-	-

Total	*Maternal near miss: mortality ratio: *MNM: 1 MD = 5.2: 1					
Non-white	*Maternal near miss: mortality ratio: *MNM: 1 MD =5.3: 1					
White	*Maternal near miss: mortality ratio: *MNM: 1 MD = 5.2: 1					

Total	*Mortality index: MI *= MD/(MNM+MD) = 0.154 = 15.4%					
Non-white	*Mortality index: MI *= MD/(MNM+MD) = 0.159 = 15.9%					
White	*Mortality index: MI *= MD/(MNM+MD) = 0.160 = 16.0%					

Total	*Maternal mortality ratio: MMR* ^*a*^= MD/LB X100.000** = **170.4/100 000 LB					

LB: 82,144; Deliveries: 82,388; a: indicators cannot be estimated for categories of skin color because this information was not available for the total number of livebirths; b: *p=0.044 (white X non-white)*

PLTC: potentially life-threatening condition; MNM: maternal near miss; MD: maternal death; MI: Mortality Index is the proportion of women with near miss who died; SMOR: Severe Maternal Outcome Ratio is the proportion of all women delivering a live newborn who had a maternal near miss event or died

**Table 2:** Distribution of pregnancy outcomes according to major obstetric complications by skin color groups.

Cause^*∗*^	PLTC n (%)	MNM n (%)	MD n (%)	p^*∗∗*^
*Hemorrhage *	1532 (23.7)	253 (41.6)	29 (25.0)	*0.009*
Non-white	676 (44.1)	112 (44.3)	14 (48.3)	*<0.001*
White	856 (55.9)	141 (55.7)	15 (51.7)	*<0.001*
*Hypertension *	4550 (70.4)	263 (43.2)	30 (25.9)	*<0.001*
Non-white	2927 (64.3)	141 (53.6)	16 (53.3)	*<0.001*
White	1623 (35.7)	122 (46.4)	14 (46.7)	*<0.001*
*Infection*	47 (0.7)	36 (5.9)	5 (4.3)	*<0.001*
Non-white	19 (40.4)	24 (66.7)	4 (80.0)	*<0.001*
White	28 (59.6)	12 (33.3)	1 (20.0)	*<0.001*
*Clinical /Surgical*	605 (9.3)	151 (24.8)	58 (50.0)	*<0.001*
Non-white	310 (51.2)	64 (42.4)	25 (43.1)	*<0.001*
White	295 (48.8)	87 (57.6)	33 (56.9)	*<0.001*
*More than one*	319 (4.9)	92 (15.1)	4 (3.4)	*<0.001*
Non-white	168 (52.7)	50 (54.3)	3 (75.0)	*<0.001*
White	151 (47.3)	42 (45.7)	1 (25.0)	*<0.001*
*Total*	*6,465*	*608*	*116*	

PLTC: potentially life-threatening condition; MNM: maternal near miss; MD: maternal death

^**∗**^They are not mutually exclusive; the sum of categories for each group can be higher than a 100%

^**∗∗**^p-values comparing the proportions among groups adjusted for the effect of cluster design using *χ*2 or exact tests

P-values in italic mean they are statistically significant

**Table 3:** Previous maternal conditions according to outcome of maternal complications by skin color group.

Previous conditions	Non-white women		P^*∗*^	White women		p^*∗*^
	PLTC	SMO		PLTC	SMO	
Any of below	1677 (48.5)	153 (48.9)	0.936	1393 (54.8)	181 (52.8)	0.592
Chronic Hypertension	646 (18.7)	39 (12.5)	*0.019*	429 (16.9)	51 (14.9)	0.491
Obesity	922 (26.7)	50 (16.0)	*0.024*	802 (31.6)	49 (14.3)	*<0.001*
Low weight	8 (0.2)	0	0.444	10 (0.4)	6 (1.7)	*0.014*
Diabetes	74 (2.1)	9 (2.9)	0.434	67 (2.6)	18 (5.2)	0.076
Smoking	175 (5.1)	22 (7.0)	0.340	198 (7.8)	28 (8.2)	0.846
Cardiac disease	89 (2.6)	17 (5.4)	*0.025*	69 (2.7)	18 (5.2)	*0.030*
Respiratory disease	72 (2.1)	10 (3.2)	0.206	106 (4.2)	20 5.8)	0.166
Renal diseases	30 (0.9)	12 (3.8)	*<0.001*	43 (1.7)	10 (2.9)	0.226
Sickle cell disease	26 (0.8)	10 (3.2)	*<0.001*	14 (0.6)	2 (0.6)	0.954
HIV/AIDS	35 (1.0)	6 (1.9)	*0.016*	32 (1.3)	8 (2.3)	0.073
Thyroid diseases	29 (0.8)	8 (2.6)	*0.009*	45 (1.8)	12 (3.5)	*0.028*
Neurologic diseases	34 (1.0)	4 (1.3)	0.653	31 (1.2)	8 (2.3)	*0.037*
Collagenoses	14 (0.4)	3 (1.0)	0.118	20 (0.8)	6 (1.7)	0.098
Cancer	7 (0.2)	2 (0.6)	0.146	9 (0.4)	6 (1.7)	*0.002*
Drug addiction	38 (1.1)	8 (2.6)	0.096	27 (1.1)	15 (4.4)	*<0.001*
Others (not specified)	132 (3.8)	37 (11.8)	*<0.001*	149 (5.9)	49 (14.3)	*<0.001*
*Total* ^*∗*^	*3458*	*313*		*2540*	*343*	

PLTC: potentially life-threatening condition; SMO: severe maternal outcomes; MNM: maternal near miss; MD: maternal death; ^*∗*^p-values comparing PLTC and SMO groups adjusted for the effect of the cluster design using *χ*^2^ or exact tests; P-values in italic mean they are statistically significant

**Table 4:** Crude estimated risks of severe maternal outcome (SMO=MNM+MD) according to sociodemographic characteristics of women by skin color.

Characteristics	Non-white women			White Women		
	SMO	PLTC	PR (95%CI)	SMO	PLTC	PR (95%CI)
*Age (years) *						
10-19	50	716	0.82 [0.60-1.12]	56	437	0.98 [0.77-1.24]
20-29	155	1786	1	169	1288	1
30-39	115	1098	1.19 [0.90-1.57]	130	805	1.20 [0.94-1.53]
40-49	24	164	*1.60 [1.10-2.33]*	25	121	*1.48 [1.04-2.11]*
*Marital status* ^*a*^						
With partner	193	1849	1	232	2380	1
Without	87	1585	*0.55 [0.36-0.83]*	116	1046	0.69 [0.44-1.10]
*Schooling* ^*b*^						
No/Primary	118	1526	0.92 [0.36-2.36]	131	872	0.81 [0.47-1.37]
High	92	1334	0.83 [0.35-1.93]	110	1068	*0.58 [0.36-0.92]*
University	11	130	1	36	186	1
*BMI* ^*d*^						
Low weight	22	251	0.80 [0.44-1.44]	57	245	1.38 [0.91-2.08]
Normal	49	435	1	76	478	1
Overweight	28	475	*0.55 [0.34-0.90]*	34	427	*0.54 [0.40-0.73]*
Obesity	26	543	*0.45 [0.26-0.78]*	31	508	*0.42 [0.27-0.65]*
*Total*	*344*	*3764*		*380*	*2651*	

PLTC: potentially life-threatening condition; MNM: maternal near miss; MD: maternal death; PR_adj_= prevalence ratio adjusted for the effect of the cluster design

Missing information for: ^a^ 394+257, ^b^ 897+628, ^d^ 2279+1175 cases; PR in italic mean they are statistically significant

**Table 5:** Crude estimated risks of severe maternal outcome (SMO=MNM+MD) according to some obstetric characteristics of women by skin color.

Characteristics	Non-white women			White Women		
	SMO	PLTC	PR (95%CI)	SMO	PLTC	PR (95%CI)
*Coverage for PN* ^*a*^						
Public	274	2932	0.85 [0.28-2.60]	277	2030	0.63 [0.39-1.03]
Private	4	65	0.58 [0.15-2.24]	7	72	0.47 [0.15-1.42]
Social security	4	36	1	28	120	1
No PN care	21	192	0.99 [0.34-2.82]	25	131	0.85 [0.31-2.34]
*Parity* ^*b*^						
0	111	1861	1	158	1280	1
1-2	149	1385	*1.73 [1.35-2.21]*	165	1042	*1.24 [1.07-1.45]*
≥3	79	511	*2.38 [1.71-3.32]*	55	324	1.32 [0.88-1.97]
*Prenatal care (visits)* ^*c*^						
No	28	257	*1.86 [1.18-2.92]*	36	153	*2.31 [1.15-4.65]*
1-5	122	1243	*1.69 [1.35-2.10]*	112	777	*1.53 [1.20-1.96]*
6 or more	93	1663	1	123	1370	1
*Gestational age at termination of pregnancy* ^*d*^						
< 22 weeks	26	177	*4.06 [1.83-8.99]*	42	165	*3.67 [1.80-7.48]*
22-27	32	169	*5.04 [2.88-8.83]*	51	164	*4.29 [2.45-7.50]*
28-33	84	684	*3.46 [2.42-4.95]*	82	500	*2.55 [1.64-3.96]*
34-36	60	754	*2.33 [1.43-3.80]*	62	462	*2.14 [1.42-3.22]*
≥ 37	58	1779	1	71	1212	1
Still pregnant	74	114	*12.47 [8.05-19.30]*	58	86	*7.28 [4.02-13.17]*
*Delays* ^*e*^						
With any delay	237	1961	*1.97 [1.45-2.69]*	240	1112	*2.25 [1.69-3.00]*
No delay	93	1609	1	124	1447	1
*Total*	*344*	*3764*		*380*	*2651*	

Missing information for: ^a^ 580+341; ^b^ 12+7; ^c^ 702+460; ^d^ 97+76; ^e^ 208+108 cases

PR_adj_= prevalence ratio adjusted for the effect of the cluster design

PR in italic mean they are statistically significant

**Table 6:** Crude estimated risks of severe maternal outcome (SMO=MNM+MD) according to pregnancy termination and perinatal outcomes by skin color.

Characteristics	Non-white women			White Women		
	SMO	PLTC	PR (95%CI)	SMO	PLTC	PR (95%CI)
*GA at delivery* ^*a*^						
Preterm (<37 weeks)	176	1448	*2.67 [1.93-3.71]*	190	1017	*2.12 [1.54-2.93]*
Term	82	1942	1	107	1336	1
Still pregnant	28	206	*2.95 [1.76-4.97]*	43	206	*2.33 [1.24-4.38]*
*Mode of pregnancy termination* ^*b*^						
Vaginal birth	70	805	1	63	749	1
Cesarean section	220	2554	0.99 [0.64-1.53]	238	1540	1.73 [0.88-3.37]
Abortion/ectopic	24	186	1.43 [0.56-3.64]	32	152	2.24 [0.79-6.34]
Still pregnant	28	206	1.50 [0.90-2.50]	43	208	2.21 [0.88-5.55]
*Apgar score *5^*th*^* min*^*c*^						
<7	27	87	*4.71 [3.28-6.77]*	31	80	*3.22 [2.24-4.63]*
≥7	164	3098	1	199	2098	1
*Birth weight* ^*d*^						
<2.500 g	136	1227	*2.33 [1.69-3.22]*	148	841	*1.99 [1.38-2.87]*
≥2.500 g	92	2058	1	114	1403	1
*Neonatal condition at birth* ^*e*^						
Live birth	204	3212	1	249	2205	1
Still birth	53	107	*5.55 [3.88-7.94]*	34	75	*3.07 [1.88-5.04]*
*Neonatal outcomes* ^*f*^						
Discharge	118	2449	1	149	1586	1
Admitted or transferred	72	628	*2.24 [1.56-3.20]*	79	538	*1.49 [1.02-2.17]*
Neonatal death	8	65	*2.38 [1.52-3.75]*	14	67	2.01 [0.98-4.15]
*Total*	*344*	*3764*		*380*	*2651*	

Missing information for: ^a^ 226+132; ^b^ 15+6; ^c^ 732+623; ^d^ 595+525; ^e^ 532+468; ^f^ 768+598 cases

PR_adj_= prevalence ratio adjusted for the effect of the cluster design; PR in italic mean they are statistically significant

**Table 7:** Conditions associated with SMO (MNM or MD) as outcome of pregnancy with severe maternal morbidity (multiple analysis by Poisson regression^*∗*^).

Model/ Condition	PR_adj_	95% CI	p
*Model [n = 4,981] for Severe Maternal Outcome*			
Other conditions	2.53	*2.09–3.05*	*<0.001*
Any delay	1.72	*1.44–2.05*	*<0.001*
Gestational age at admission (<37 weeks or postpartum)	2.83	*1.99–4.01*	*<0.001*
Obesity	0.48	*0.37–0.62*	*<0.001*
Number of previous deliveries (≥1)	1.33	*1.10–1.62*	* 0.005*
*Skin color/ethnicity (non-white)*	0.61	*0.44–0.85*	* 0.005*
Marital status (Without partner)	0.56	*0.38–0.83*	* 0.005*
Renal diseases	1.89	*1.16–3.05*	* 0.012*
Neoplasms	1.79	*1.09–2.96*	* 0.024*
Sickle cell diseases	2.01	*1.09–3.69*	* 0.026*
Schooling (up to primary)	1.22	*1.02–1.47*	* 0.033*
Chronic hypertension	0.71	*0.51–0.99*	* 0.049*

^*∗*^Analysis considering cluster design (center)

Predictors entering the models: age (years); marital status (with partner: 0/ without: 1); schooling (up to primary: 1/ high school or higher: 0); skin color/ethnicity (White: 0/ non-white: 1); financial support for prenatal care (public: 0/ other: 1); number of previous deliveries (0/ ≥1: 1); number of prenatal visits (< 6: 1/ ≥6: 0); gestational age at admission (< 37 weeks or postpartum: 1/ ≥37 sem.: 0); any delay (Yes: 1/ No: 0); previous pathological conditions (Yes: 1/No: 0); chronic hypertension (Yes: 1/No: 0); obesity (Yes: 1/No: 0); low weight (Yes: 1/No: 0); diabetes (Yes: 1/No: 0); smoking (Yes: 1/No: 0); cardiac diseases (Yes: 1/No: 0); respiratory diseases (Yes: 1/No: 0); renal diseases (Yes: 1/No: 0); sickle cell diseases (Yes: 1/No: 0); HIV/Aids (Yes: 1/No: 0); thyroid diseases (Yes: 1/No: 0); neurological diseases/epilepsy (Yes: 1/No: 0); collagenoses (Yes: 1/No: 0); neoplasms (Yes: 1/No: 0); drug addiction (Yes: 1/No: 0); other conditions (Yes: 1/No: 0).

BMI was not included in the models due to the high number of missing values

## Data Availability

The data used to support the findings of this study are available from the corresponding author upon request.

## Abbreviations

CDC: Center for Disease Control and Prevention
LB: Live births
MD: Maternal death
MI: Mortality Index is the proportion of women with near miss who died
MNM: Maternal near miss
MD: Maternal death
PLTC: Potentially life-threatening condition
PR_adj_: Prevalence ratio adjusted for the effect of the cluster design
SMO: Severe maternal outcomes
SMOR: Severe maternal outcome ratio
WHO: World Health Organization.
